# Efficacy of Body Representation Rehabilitation Training for Adults with Unilateral Brain Damage: A Preliminary Study

**DOI:** 10.3390/brainsci15020140

**Published:** 2025-01-30

**Authors:** Maria Cropano, Mariachiara Gaita, Erica Dolce, Silvia Canino, Valentina Gerarda Angelillo, Antonella Di Vita, Maddalena Boccia, Simona Raimo, Liana Palermo

**Affiliations:** 1 Department of Health Sciences, Magna Graecia University of Catanzaro, 88100 Catanzaro, Italy; 2 UOSD Second Neurology, University of Campania Luigi Vanvitelli, 80131 Naples, Italy; 3 Department of Psychology, University of Campania Luigi Vanvitelli, 81100 Caserta, Italy; 4 Department of Medical and Surgical Sciences, Magna Graecia University of Catanzaro, 88100 Catanzaro, Italysimona.raimo@unicz.it (S.R.); 5 Rehabilitation Clinic ‘Villa delle Magnolie’, 81020 Castelmorrone, Italy; 6 Department of Human Neuroscience, Sapienza University of Rome, 00185 Rome, Italy; 7 Department of Psychology, Sapienza University of Rome, 00185 Rome, Italy; 8 Cognitive and Motor Rehabilitation and Neuroimaging Unit, IRCCS Santa Lucia, 00175 Rome, Italy

**Keywords:** body representation, body awareness, body schema, rehabilitation, unilateral brain damage, stroke

## Abstract

Background/Objectives: Body representations (BRs) are essential for guiding movements, maintaining spatial awareness, and achieving effective interactions with the environment. Several studies suggest that BRs are frequently impaired following unilateral brain damage, emphasising the need for tailored rehabilitation interventions; however, there is a lack of studies evaluating the effectiveness of training specifically designed to improve different kinds of functional BRs after stroke. Therefore, the present study aimed to present and implement a specific rehabilitation training program for BR alterations and evaluate its effectiveness in a sample of adults with unilateral brain damage. Methods: Nine adults with unilateral brain damage and seven age- and education-matched healthy controls were recruited. Both groups underwent a neuropsychological assessment to evaluate BR (action- and nonaction-oriented). Additionally, functional autonomy and motor functioning were assessed in the patient group. Following an initial assessment (T0), the patients participated in a BR-specific rehabilitation intervention. At the end of the rehabilitation program (T1), both groups were re-evaluated with the same tasks used at T0. Results: At T0, the patient group performed worse on BR tasks than the controls. At T1, a significant improvement in the nonaction-oriented BR and functional autonomy was observed in the patient group. Conclusions: This preliminary study suggests the effectiveness of a targeted rehabilitation intervention for BR in promoting enhanced body boundary awareness and greater accuracy in the perception of body part positions, possibly leading to increased functional autonomy. These findings highlight the importance of incorporating BR training in rehabilitation programs for adults with acquired brain damage, alongside motor rehabilitation.

## 1. Introduction

Body representation (BR) is a dynamic process requiring the synthesis of motor and sensory inputs, including visual, proprioceptive, and interoceptive information. This integration enables the brain to maintain an updated neural model of the body, which supports motor coordination and self-perception [1,2,3].

Numerous BR theoretical models have been proposed, but a universally accepted taxonomy remains elusive [3,4,5]. However, scientific evidence from neuroimaging and neuropsychological studies [6,7] underscores the clinical and functional significance of distinguishing between action-oriented BR (aBR), also known as body schema, and nonaction-oriented BR (naBR). In particular, aBR refers to dynamic mental representation critical for action and interaction with the environment, integrating multiple sensory and motor inputs. In contrast, naBR is critical for perception, recognition, body ownership, and self-awareness, and includes the visuospatial body map, which organises visual information about body part boundaries and spatial relationships [8]. These BRs appear to engage distinct neural substrates. For instance, aBR is associated with activation in the primary motor cortex and the right extrastriate body area, whereas naBR is more closely linked to activity in the primary somatosensory cortex and the supramarginal gyrus [6].

Studies that have systematically examined the different BRs in adults with brain damage report the presence of deficits in at least one BR in 51% of patients with unilateral brain damage [8] and in 81% of patients when both bilateral and unilateral brain damage are considered [9], pointing to the extensive occurrence of BR deficits after stroke. In these studies, however, the authors have not evaluated the presence of deficits in cognitive functions unrelated to body processing that could affect BR (e.g., language or visuospatial abilities). Recently, Raimo and colleagues [10] overcame this limitation, conducting a study to assess the impact of left or right unilateral brain damage on different BRs, using a specific battery of BR tests that also included control tasks with stimuli unrelated to the body in order to control for potential influences of cognitive abilities required to perform the BR tasks. In this study, approximately 64% of adults with unilateral brain damage showed an impaired performance in at least one of the tasks exploring the BR; also, approximately 37.5% of them showed a selective BR deficit when considering the performance in the cognitive control tasks, suggesting that BR deficits in adults with unilateral brain damage are likely not the result of a general impairment in cognitive functioning.

Any deficits in aBR or naBR after a stroke are the direct consequence of damage in the brain areas that are relevant for building efficient BRs [11,12,13]. Also, post-stroke motor deficits impair movement and interaction with objects, which, in turn, may further disrupt the BR of the affected body part, exacerbating disability and motor difficulties [14].

Taken together, these findings underline the importance of a detailed exploration of BRs in routine neurological and neuropsychological evaluation (see also [15]). Indeed, such deficits could affect motor outcomes and daily functioning in adults with unilateral brain damage, highlighting the need to implement specific rehabilitation protocols to address these impairments.

Despite being widespread and significantly impacting the quality of life, few studies have specifically and systematically investigated the rehabilitation of different BR deficits in adults with unilateral brain damage [16]. Also, so far, most rehabilitation protocols have indirectly targeted BRs by focusing on the altered inflow of sensory, proprioceptive, and/or motor information crucial to building BRs. In this vein, various rehabilitation approaches, such as mirror therapy and other crossmodal illusions, action observation, virtual reality, and neurorobotics [17,18,19], have been proposed to facilitate relearning of motor skills through intensive, repetitive training. In turn, it has been suggested these rehabilitation protocols can be useful for improving BR deficits [14,19] based on the idea that providing the missing somatosensory information could modulate the distorted BRs. However, few studies have directly tested this hypothesis in adult patients with brain damage (for mirror box therapy, see [14]; for neurorobotics, see [20]).

Also, no comprehensive, specialised training protocol for the rehabilitation of aBR and naBR itself in adults with brain damage has been developed. Therefore, the present study aims to present a novel rehabilitation training program focused specifically on functional BRs and to evaluate its effects on improving BRs and functional outcomes in adults with unilateral brain damage. This research contributes to a growing body of literature on BR by offering insights into its role in optimising therapeutic strategies and designing targeted rehabilitation interventions.

## 2. Materials and Methods

### 2.1. Participants

Nine participants with unilateral brain damage (4 with left brain damage and 5 with right brain damage) and seven age- and education-matched healthy controls were enrolled in the study. Adults with unilateral brain damage were recruited at the Rehabilitation Clinic, “Villa delle Magnolie” (Caserta, Italy). Exclusion criteria included multiple cerebrovascular incidents, neoplastic or traumatic causes, cognitive deterioration, psychiatric illnesses, substance abuse, or significant language comprehension deficits. Demographic information and neuropsychological data for patients with unilateral brain damage are shown in Table 1. Healthy controls were recruited from the local community of Catanzaro (Italy) and were included only if they had no history of neurological or psychiatric disorders, cognitive impairments, or substance abuse.

The research adhered to the ethical principles outlined in the Declaration of Helsinki and received approval from the local Ethical Committee. All participants provided informed written consent.

### 2.2. Neuropsychological Evaluation at T0

All participants underwent a first neuropsychological assessment (T0) that included:

- the hard copy version of the Mini-Mental State Examination (MMSE [21,22]) to assess global cognitive functioning. It explores seven different cognitive areas: orientation in time and space, word registration, attention and calculation, recall, language, and constructive praxis. The total score ranges from 0 to 30 points;
- the web-based version of the Hand Laterality Task (wHLT [23,24]) to assess aBR (Body Schema). Participants were asked to evaluate the laterality of a target hand (24 left and 24 right hands) displayed on the top of the screen with eight different angular rotations (0° to 315°). Participants had to rotate each hand mentally and then indicate their answer by selecting the left or the right hand shown at the bottom of the screen, not rotated. Similarly, in the corresponding control task, the Object Laterality Task (OLT), participants were asked to determine the laterality of a flower (24 flowers with a leaf positioned at the left of the stem and 24 flowers with a leaf positioned at the right of the stem) presented with the same angular variations of the HLT by selecting one of the two response options at the bottom of the screen (i.e., a flower with a leaf positioned at the left of the stem and a flower with a leaf positioned at the right of the stem but not rotated). Each task comprised 48 items and four practice items to ensure comprehension. One point was assigned for each correct response, with higher scores indicating better performance (maximum score: 48). The task order was counterbalanced, and accuracy and reaction times were recorded;
- the web-based Frontal Body Evocation Task (wFBET [23,24]) to assess naBR. Participants were asked to view the drawing of a body for 10 s and then to determine whether a body part (e.g., right or left hand, arm, leg, and foot) was correctly or incorrectly positioned with respect to another body part (i.e., the torso or the head) acting as a reference point. Each body part was presented six times in three variations: correct, incorrect with a minimal deviation from its proper location, and incorrect with a significant deviation from its proper location. All variations included 16 stimuli—8 with the torso as the point of reference and 8 with the head as the point of reference. Similarly, in the corresponding control task, the web-based Christmas Tree Task (wCTT), participants were asked to view a picture of a Christmas tree for 10 s and determine whether a part of the tree (i.e., right or left lower branches, middle branches, upper branches on the left or right and middle branch) was correctly or incorrectly positioned with respect to two different reference points (the star tree topper or the pot). Each part was presented six times in three variations as for the wFBET. Each task comprised 48 items. One point was assigned for each correct response, with higher scores indicating better performance (maximum score: 48). The task order was counterbalanced, and accuracy and reaction times were recorded.

In addition, the following tests and questionnaires were administered to participants with unilateral brain damage:

- the Token Test [25], used to evaluate oral language comprehension. This test was administered only to patients with left unilateral brain damage to exclude severe language comprehension impairment;
- the Standard Battery for the Evaluation of Hemineglect [26] and the Use of Common Objects Test [27], used only for patients with right unilateral brain damage to assess the presence of extrapersonal and personal neglect;
- the Raven’s Coloured Progressive Matrices (RCPM [28]) to exclude the presence of a deficit in abstract reasoning;
- the Rivermead Mobility Index (RMI [29]) to evaluate functional mobility, including gait, balance, and transfers. This index comprises 15 items: 14 self-reported and one directly observed. The items are scored 0 if the patient is not able to complete the task or 1 if they are able to complete it. The total score ranges from 0 to 15, with higher scores stipulating better functional mobility;
- the Barthel Index (BI [30]) to assess functional disability across 10 domains: feeding, bathing, grooming, dressing, bowel and bladder control, toileting, chair transfers, ambulation, and stair climbing. Items are weighted based on the level of nursing care required and scored as 10 (independent), 5 (some assistance), or 0 (dependent). The total score ranges from 0 to 100, with higher scores reflecting greater functional autonomy. Participants with unilateral brain damage were tested in a quiet, dedicated room at the rehabilitation clinic, while healthy participants were tested at the Laboratory of Cognitive Processes at Magna Graecia University of Catanzaro. The testing session lasted approximately 60 min, providing flexibility for participants to complete all tasks at their own pace.

### 2.3. BR Rehabilitation Training

#### 2.3.1. Background and Rationale

To the best of our knowledge, at the time we designed this study, there were no published rehabilitation protocols specifically targeting aBR and naBR. All the material (i.e., pictures, videos, etc.) was specifically created for this study by our research team, but the rationale for the exercises was derived from earlier experimental tasks developed to assess aBR or naBR (e.g., [8,9]), as well as from previous action observation training protocols (e.g., [31]). Although those protocols were not originally designed to address BR deficits, theoretical studies have suggested a tight interplay between aBR and action observation (e.g., [32]).

The training aimed at improving: i. the aBR (body schema) through the real or imaginary (motor imagery) execution of actions and the observation of actions involving the use of the body; and ii. the naBR through exercises aimed at enhancing the ability to analyse the spatial relations between the different body districts.

Specifically, the aBR exercises were based on two premises: (i) that actual and mentally simulated movements depend on aBR (indeed, mental rotation of body parts is the more common measure to assess aBR [5,6,7,8,9,10,15]); and (ii) that since the repeated practice of an activity should promote neuroplasticity—reinforcing the neural connections underlying the task and, in turn, improving the ability to perform it [33]—the repeated use of aBR to perform real or imaged movements/actions should lead to an improvement of the aBR difficulties.

The naBR exercises were designed to improve the ability to process structural and perceptual features of the human body and, in particular, first-order relational information (e.g., arms are attached to the upper torso) and the relative size of body parts that are relevant information for building naBR [34].

Examples of the stimuli for the exercises of the BR rehabilitation training, along with the instructions, are reported in Supplementary Materials S1 and are briefly presented in the following sections.

#### 2.3.2. General Procedure

Only the group of participants with unilateral brain damage underwent rehabilitation training specifically designed to improve aBR and naBR.

The training was carried out within 5 days after the baseline assessment to ensure that everyone was retested after the same interval, maintaining consistency in the evaluation process while also accommodating organisational constraints. This was consistent with previous rehabilitation studies (e.g., [35,36,37,38]). The rehabilitation protocol involved two 40 min (see [39,40,41,42]) training sessions per week over a period of six weeks.

The exercises of the training sessions for aBR and naBR were presented in a fixed sequence to maintain consistency across all participants.

#### 2.3.3. Exercises for aBR

*Real rotations* (adapted from the assessment of aBR in [8,9,10]): the material included photos of body parts in various positions. The patient was asked to position his body in the same way as represented in a photo (e.g., positioning the foot rotated 45° towards the right). For each session, this exercise was proposed 4 times.

*Action Observation* (adapted from training exercises in [31]): the material included a set of 24 videos paired with a questionnaire. Participants viewed videos of others performing actions. To verify attention, they were asked a question about the observed action after watching. Each session required participants to observe two different actions.

*Motor Imagery:* This exercise was based on imagery questionnaires (e.g., [43]) and on the idea that motor imagery as real actions requires aBR (see [8,9,10]). The material included a set of 24 actions paired with a questionnaire designed to engage motor imagery. Participants were asked to imagine themselves performing a given action. To ensure active engagement, a follow-up question verified their use of motor imagery. For instance, after imagining making an “OK” gesture with their fingers, the participant might be asked which fingers touch during this action (correct answer: thumb and index finger). Each session required participants to imagine two different actions.

*Judgments of body actions:* This exercise was based on gesture recognition tasks (see [44]). The material included 11 pairs of photos depicting actions with body parts positioned correctly/incorrectly for the action. For each action, two photos (correct and incorrect) were presented, and the patient had to identify the correct image. For the image containing the error, the patient was required to point out where the error was located. Two actions per session were presented.

#### 2.3.4. Exercises for naBR

*Puzzles:* This exercise was designed to improve the ability to process first-order relational information among body parts and was inspired by tasks used in previous studies to assess naBR in patients with autotopagnosia [45] in which a nonaction-oriented BR deficit has been described [7]. The materials comprised 12 puzzles of varying difficulty levels, each depicting human figures. The difficulty was determined by the number of pieces (6–12) and whether a reference model (a complete photograph of the person) was provided or not. During each session, the participant reconstructed one puzzle, progressing to increasingly complex levels.

*Human Body with Misplaced or Disproportionate Parts:* This exercise was inspired by experimental tasks used in previous studies to assess naBR and, in particular, first-order relational information among body parts and body part proportions (e.g., [34,46]). The materials included images depicting either correctly or incorrectly represented body parts. Errors could involve improper positioning or disproportions. Participants were asked to determine whether each image was correct or incorrect. For incorrect images, they identified the specific error. For each session, four images were presented.

*Wooden Mannequin Exercises* (adapted from the assessment of naBR in [8,9])*:* the material included two wooden mannequins and a list of postures. Two variations of this task were conducted:

(a) The therapist positioned the mannequin in a specific posture (e.g., touching its face with one hand), and the participant mimicked the pose.
(b) Two mannequins were displayed in different positions (e.g., both with their left arm raised). The participant determined whether the two mannequins were positioned identically. For each session, this exercise was performed four times.

*The body dominoes*: This exercise was adapted from a traditional board game and developed to enhance the understanding of body part proximity and the relationships between body parts. The choice of adapting a well-known board game was informed by the literature emphasising the benefits of gamification in rehabilitation settings for enhancing engagement (e.g., [47]).

The material consists of tiles divided into two square ends, each containing an image or a name of body parts. The following sets of tiles were available: 32 with two names of body parts, 32 with two images of body parts, and 32 with one name and one image of a body part. Tiles were distributed between the patient and the therapist, who took turns placing them on the table. The patient/therapist could place one of their tiles next to those already on the table only if it featured a body part contiguous to one indicated at the edges of the tiles on the table. For each session, this exercise was performed one time.

### 2.4. Neuropsychological Evaluation at T1

After 6 weeks, both the participants with unilateral brain damage and the healthy controls underwent a second neuropsychological evaluation (T1) with the same tasks and questionnaires used in T0. Participants with unilateral brain damage underwent the assessment within five days after the end of the BR rehabilitation training specifically designed for them. The control group, who did not receive any training, underwent retesting at the same time intervals as the patients. The retest (T1) of the control group was relevant to evaluate potential learning effects associated with the testing materials.

### 2.5. Statistical Analysis

Non-parametric statistical analyses were chosen due to the small sample size, which limited the possibility of meeting the assumptions required for parametric tests.

The Mann–Whitney U test was used to compare demographic and neuropsychological variables between the participants with unilateral brain damage and the healthy participants at T0.

The Wilcoxon signed-rank test was performed to assess improvements in BR, functional autonomy, and motor outcomes following the BR rehabilitation training in the participants with unilateral brain damage, analysing performance changes between the two neuropsychological assessments (T0 and T1), with the assumption of better performance at T1 than at T0 (one-tailed). Additionally, the Wilcoxon signed-rank test was applied to the healthy controls to determine whether changes in BR occurred independently of the BR rehabilitation training effect by comparing their performance at T0 and T1, assuming better performance at T1 than at T0 (one-tailed).

Multiple comparison issues were accounted for by controlling the False Discovery Rate (FDR) at 0.05, using the Benjamini–Hochberg method [48].

Finally, only for the BR tasks in which the Wilcoxon signed-rank test showed a significant improvement in patients (and for the relative control tasks, to take into account possible spurious effects), a relative functional gain score (RFG) was calculated for each participant in both the patient and the control groups (see [31,49,50]). The RFG score is a measure of improvement that adjusts for the score at baseline and reflects the proportion of potential improvement achieved during training, and it was calculated as follows:

RFG = [test score at T1-test score at T0]/[maximum score-test score at T0].

Then, the Mann–Whitney U test was used to compare the two groups, assuming greater improvement in the patient group that underwent the training (one-tailed).

## 3. Results

### 3.1. Comparison Between Participants with Unilateral Brain Damage and Healthy Controls at T0

The group of patients with unilateral brain damage and the group of healthy controls did not differ in age (Mann–Whitney U = 29.5, *p* = 0.837) and education (Mann–Whitney U = 19, *p* = 0.21). See Table 2. This was a critical prerequisite since these demographic variables could affect cognitive performance.

Concerning cognitive performance (see Table 3), at T0, the two groups showed comparable levels of global cognitive functioning (Mann–Whitney U = 11.5, *p* = 0.181, FDR-adj *p* = 0.181). In the tasks assessing BR and the corresponding control tasks, the Mann–Whitney U test showed that the patients with unilateral brain damage performed significantly worse than the healthy controls in the wFBET (Mann–Whitney U = 2, *p* = 0.001, FDR-adj *p* = 0.003), in the wCTT (Mann–Whitney U = 3.5, *p* = 0.001, FDR-adj *p* = 0.003), and in the wHLT (Mann–Whitney U = 10, *p* = 0.023, FDR-adj *p* = 0.038). The two groups showed similar performance in the wOLT (Mann–Whitney U = 18.5, *p* = 0.174, FDR-adj *p* = 0.181).

### 3.2. Comparison Between T0 and T1 in the Group of Participants with Unilateral Brain Damage and in the Group of Healthy Controls

Conducted in order to compare performance at T0 and T1 in the group of participants with unilateral brain damage, the Wilcoxon signed-rank test showed a marginally significant improvement at T1 in the wFBET (Z = −2.37, *p* = 0.009, FDR-adj *p* = 0.051, one-tailed) and in the BI (Z = −2.12, *p* = 0.017, FDR-adj *p* = 0.051, one-tailed) when controlling the FDR at 0.05 using the Benjamini–Hochberg method. As shown in Table 4a, no significant differences between T0 and T1 were observed for the wCTT, wHLT, wOLT, or RMI when controlling the FDR. Mean scores and standard deviations of BR and control tasks, functional autonomy, and motor functioning questionnaires for the patient group are reported in Table 4a.

As shown in Table 4b, the Wilcoxon signed-rank test conducted to compare performance at T0 and T1 in the group of healthy controls showed no significant improvements at T1 in wFBET, wCTT, wHLT, and wOLT. Mean scores and standard deviations of BR and control tasks for the healthy controls are reported in Table 4b.

Finally, the Mann–Whitney U tests that were applied exclusively to the BR tasks where the Wilcoxon signed-rank test indicated a significant improvement at T1 in patients, as well as the corresponding control tasks to account for potential spurious effects, showed a greater improvement in the patient group (mean RFG = 4.84, SD = 5.13) than in the control group (mean RFG = 0.42, SD = 3.81) in the wFBET (U = 14, *p* = 0.036, one-tailed). No significant differences between the patient (mean RFG = 2.44, SD = 5.15) and the healthy control group (mean RFG = 0.52, SD = 2.87) were found in the wCTT (U = 24, *p* = 0.235, one-tailed).

## 4. Discussion

The present study aimed to present a new rehabilitation training program focused on BRs and to preliminarily evaluate its effectiveness in adults with unilateral brain damage.

First, we found that before the rehabilitation training program, patients with unilateral brain damage, compared with healthy controls, exhibited significantly poorer performance on aBR and naBR tasks. This result aligns with the existing literature [8,9,10,11,12] that highlights the vulnerability of BR following unilateral brain damage. However, a significant difference was also observed in the control task of naBR (wCTT), which was designed to assess general visuospatial and motor-related processing unrelated to BR [10,23,24]. This finding suggests that the observed impairments in the patient group may not be limited to BR but could also reflect broader deficits in visuospatial processing and attention.

Second, we found that the BR rehabilitation training program is effective for adults with unilateral brain damage in improving naBR and functional autonomy. Considering the absence of changes in the healthy controls in tasks tapping BRs, this improvement cannot be the mere consequence of the practice effect; in other words, the change in the naBR performance was not the result of retaking the same BR task. Furthermore, patients performed better after the training in the naBR task but not in the matched control task. This was confirmed by an analysis of the relative functional gains, which showed that the patients improved more than the controls in the naBR task (wFBET) but not in the matched control task (wCTT). This analysis took into account the baseline performance, suggesting that the observed effects were due to the intervention rather than pre-existing differences.

Notably, the improvements in naBR were accompanied by gains in functional autonomy, further pointing to the connection between BR and the ability to perform daily activities [51,52]. Indeed, an accurate BR allows individuals to have a clear and precise understanding of their bodies’ capabilities, limitations, and spatial relationships that could be particularly relevant in daily routines such as dressing and grooming, achieving greater functional autonomy.

These results are consistent with the idea that BRs are highly plastic [19] and underscore the importance of targeting BRs to promote post-stroke functional recovery. However, the lack of changes in aBR and motor functioning raises questions about the specificity of the intervention and the underlying neurophysiological differences. The specific improvement in naBR suggests that the rehabilitation training could only facilitate the perceptual and cognitive realignment of static BR post-stroke. These findings are consistent with previous studies showing that rehabilitation focusing on visuomotor imagery can enhance somatic awareness, reduce phenomena like unilateral spatial neglect [53,54], and support the integration of altered sensory information after stroke.

In contrast, the BR rehabilitation training did not result in improvements in aBR or motor functioning. This discrepancy could be explained by evidence that these two BRs are involved in different processes and mediated by different neural networks [6]. Moreover, at baseline, the participants with unilateral brain damage showed greater difficulties compared with the healthy adults in naBR rather than in aBR; this suggests that BR rehabilitation training might be more effective for individuals with a more severe BR deficit, as they were more impaired and thus potentially more responsive to targeted interventions. For individuals with less severe BR impairments, alternative approaches like virtual reality training [55,56] might prove more effective. These results highlight the importance of understanding the functional specialisation and adaptability of different neural networks and cognitive processes involved in BR.

This preliminary study has some limitations. The first is related to the small sample size that limits the generalizability of the findings. Additionally, non-standardised tests were used to assess BR (see for this issue [16]), and the lack of normative data prevented the identification of clinically significant BR deficits. Finally, the lack of a control group of adults with unilateral brain damage who did not undergo the BR rehabilitation training raises concerns about the internal validity of the findings and the ability to draw firm conclusions about the specific effects of the training.

Future studies should investigate the efficacy of BR rehabilitation training in larger samples of patients with unilateral brain damage—using standardised tools to evaluate aBR and naBR and incorporate a randomised controlled trial design—where participants are randomly assigned to either a training group or a control group in order to provide stronger evidence for the specific efficacy of the training program and offer more robust insights into its potential as a rehabilitative tool.

## 5. Conclusions

In conclusion, this preliminary study highlights the potential effectiveness of a targeted rehabilitation intervention for BR in improving the awareness of the borders of the body, the location of body parts, and distance relations between body parts, which may contribute to greater functional autonomy. Taken together, these results emphasise the importance of designing targeted rehabilitation interventions that address the multifaceted nature of BR. Clinically, these findings underscore the relevance of developing and utilising BR training-based rehabilitation protocols, as they may serve as valuable tools to support motor rehabilitation.

## Figures and Tables

**Table 1:** Demographic information and neuropsychological data for adults with unilateral brain damage.

Participants	Lesion Side	Age	Education	Sex	Time Post-Onset	RMI	BI	MMSE	RCPM
Pt 1	RBD	68	8	F	SA	6	25	23	13
Pt 2	LBD	53	8	M	SA	5	20	28	16
Pt 3	LBD	64	8	M	Cr	15	100	29	28
Pt 4	RBD	49	8	M	SA	4	15	27	13
Pt 5	LBD	75	11	M	SA	9	85	18	11
Pt 6	LBD	58	16	F	SA	6	10	N.A.	26
Pt 7	RBD	64	8	F	SA	4	5	25	13
Pt 8	RBD	36	8	F	Cr	N.A.	N.A.	N.A.	N.A.
Pt 9	RBD	74	5	M	Cr	N.A.	N.A.	N.A.	N.A.

Abbreviations: LBD, left brain damage; RBD, right brain damage; M, Male; F, Female; SA, subacute phase (10–90 days from stroke); Cr, chronic phase (>90 days from stroke); RMI, Rivermead Mobility Index; BI, Barthel Index; MMSE, Mini-Mental State Examination; RCPM, Raven’s Coloured Progressive Matrices; N.A., not available.

**Table 2:** Means and standard deviations of demographic information in the two groups.

	Patients with Unilateral Brain Damage (*n* = 9)	Healthy Controls (*n* = 7)		
	Mean ± SD	Mean ± SD	U	*p*
Age	60.11 ± 12.58	60.85 ± 2.41	29.5	0.837
Education	8.88 ± 3.05	10.28 ± 2.13	19	0.210
Sex (F/M)	4/5	3/4	-	-

Note: SD, Standard Deviation; F, Female participants; M, Male participants.

**Table 3:** Means and standard deviations of MMSE, BR, and control tasks in the two groups at T0.

	Patients with Unilateral Brain Damage (*n* = 9)	Healthy Controls (*n* = 7)			
	Mean ± SD	Mean ± SD	U	*p*	FDR-Adj *p* Value
MMSE	25 ± 4.04	27.85 ± 0.89	11.5	0.181	0.181
wFBET	29.11 ± 6.60	41.71 ± 4.15	2	**0.001**	**0.003**
wCTT	32 ± 6.61	43.57 ± 2.76	3.5	**0.001**	**0.003**
wHLT	35.77 ± 7.90	45 ± 3.36	10	**0.023**	**0.038**
wOLT	38.22 ± 10.68	45.28 ± 3.03	18.5	0.174	0.181

Note: SD, Standard Deviation; FDR, Benjamini–Hochberg False Discovery Rate; MMSE, Mini-Mental State Examination; wFBET, web-based Frontal Body Evocation Task; wCTT, web-based Christmas Tree Task; wHLT, web-based Hand Laterality Task; wOLT, web-based Object Laterality Task. Significant differences are in bold.

**Table 4:** Means, standard deviations, and comparisons at T0 and T1 in the two groups of participants.

**(a) Patients with unilateral brain damage (*n* = 9)**					
	**T0**	**T1**			
	**Mean ± SD**	**Mean ± SD**	**Z**	***p*** **(one-tailed)**	**FDR-adj *p* value**
wFBET	29.11 ± 6.60	34.55 ± 6.76	−2.371	0.009	0.051
wCTT	32 ± 6.61	35.11 ± 8.03	−1.673	0.047	0.071
wHLT	35.77 ± 7.90	38.22 ± 7.37	−1.278	0.101	0.101
wOLT	38.22 ± 10.68	40.77 ± 8.95	−1.362	0.087	0.101
BI	37.14 ± 38.60	52.14 ± 40.50	−2.121	0.017	0.051
RMI	7 ± 3.91	9.14 ± 3.97	−1.841	0.033	0.066
**(b) Healthy Controls (*n* = 7)**					
	**T0**	**T1**			
	**Mean ± SD**	**Mean ± SD**	**Z**	***p*** **(one-tailed)**	**FDR-adj *p* value**
wFBET	41.71 ± 4.15	43 ± 1.63	−0.854	0.197	0.306
wCTT	43.57 ± 2.76	45 ± 1.41	−1.279	0.101	0.306
wHLT	45 ± 3.36	46.28 ± 1.60	−0.509	0.306	0.306
wOLT	45.28 ± 3.03	46.71 ± 1.88	−0.736	0.231	0.306

Note: SD, Standard Deviation; FDR, Benjamini–Hochberg False Discovery Rate; wFBET, web-based Frontal Body Evocation Task; wCTT, web-based Christmas Tree Task; wHLT, web-based Hand Laterality Task; wOLT, web-based Object Laterality Task; BI, Barthel Index; RMI, Rivermead Mobility Index.

## Data Availability

The raw data supporting the conclusions of this article are available upon reasonable request from the corresponding author due to privacy concerns for the individuals who participated in the study.

## Supplementary Materials

The following supporting information can be downloaded at www.mdpi.com/article/10.3390/brainsci15020140/s, Examples and instructions of exercises for the Body Representation (BR) Rehabilitation Training.
