# Supplementary material for: Efficacy of Body Representation Rehabilitation Training for Adults with Unilateral Brain Damage: A Preliminary Study
Source: Brain Sci. 2025 Jan 30;15(2):140. doi: 10.3390/brainsci15020140 (PMC11853476; doi:10.3390/brainsci15020140)
Supplement: Supplementary file 1 [file brainsci-15-00140-s001.zip › brainsci-3429213-supplementary.pdf]

Supplementary Material 1: Examples and instructions of exercises for the Body Representation (BR) Rehabilitation Training.

All BR Rehabilitation Training exercises can be obtained upon request from the corresponding author.

Exercises for action-oriented BR

1) Real Rotations
The participant is given the following instruction: "Now I'm going to show you pictures of body parts in different rotations or positions. Your task is to replicate each position you see in the pictures."
For each session, 3 different rotations are proposed.
If the participant is unable to place their body part in the same way as depicted in the picture, the examiner can place his or her body in the same or different way as depicted in the picture and ask the participants whether the position that the examiner has assumed is the same or different from the one in the picture.

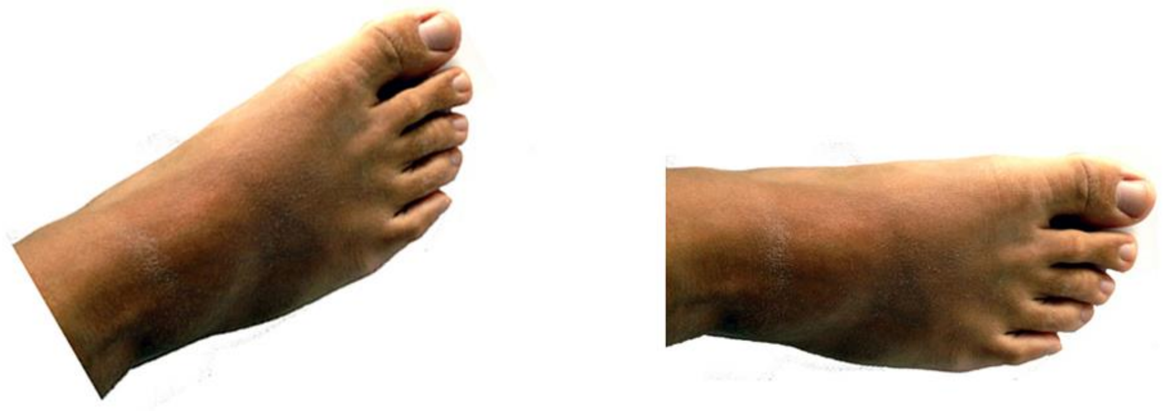

Figure S1. *Example of Real Rotation Exercise*.

2) Action Observation
The participant is given the following instruction: "I'm going to show you some videos now. Please watch them carefully, as I'll ask you some questions about them afterwards".
For each session, 2 different videos are proposed.
When showing a video, make sure to play it in a loop since the videos are short. Ensure that each video is watched at least twice.

Table S1. Example of Action Observation Exercise.

Video	Question	Answer
Moving an object horizontally	What was the man moving in the video?	A pen holder
Vertical movement	What was the man moving in the video?	A cane/stick
Standing up after kneeling	What was the man doing?	Kneeling and standing up
Kicking a ball	What was the man kicking?	A ball

3)Motor Imagery

The participant is given the following instruction: “Now, I’d like you to close your eyes and imagine yourself performing actions that involve using your body. Afterward, I’ll ask you a question about the action you imagined”. For each session, 2 different actions are proposed.

Table S2. Example of Motor Imagery Exercise.

**Action**	**Question**	**Answer**
Imagine you are making the OK sign (with your right hand/sn).	Which fingers touch when you make the OK sign?	Thumb and index finger
Imagine making the sign that you are mad (with your right hand/sn).	What part of your body do you touch to make the sign that you are crazy?	Head
Imagine you are riding a bicycle.	What do you move mainly?	Legs, feet
Imagine you are kneeling on the floor.	Once you are on your knees, does the sole of your foot (the bottom part of your foot) touch the floor?	No

4) Judgments of BodyActions

The participant is given the following instruction: “I am now showing you two pictures of an action. Please point to the picture that shows the action performed correctly”.
For each session, 2 different actions are proposed.

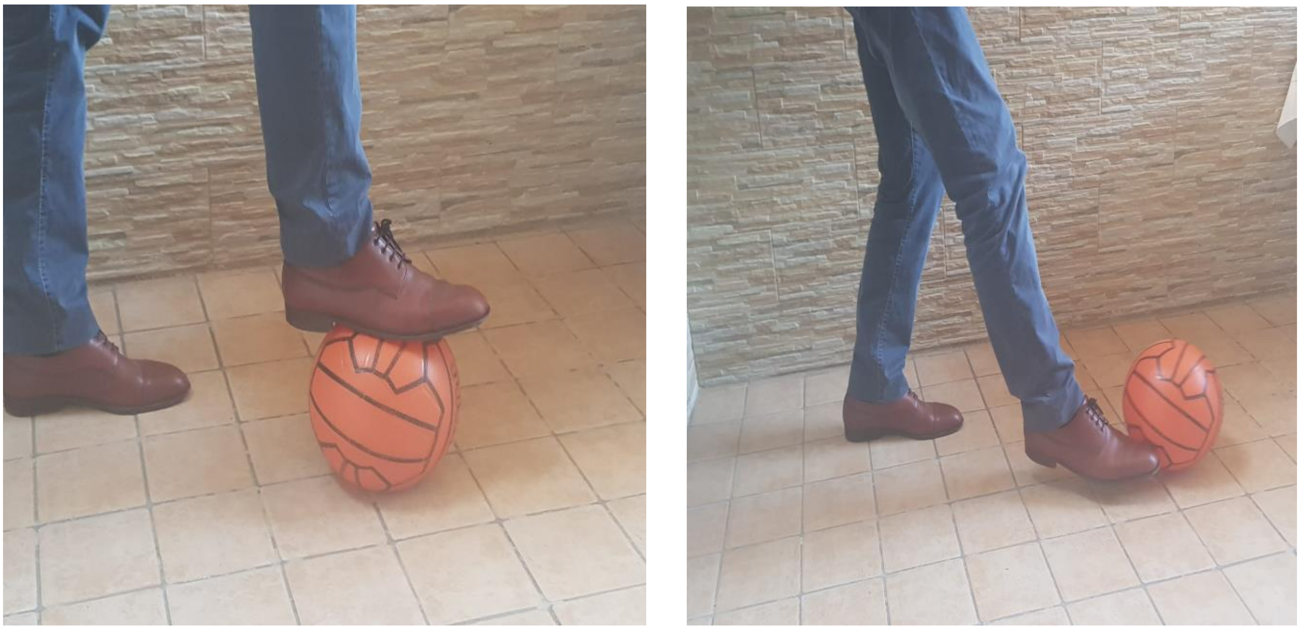

Figure S2. *Example of Judgments of Body Actions Exercise.*

In this example, the participant is asked: In which picture does the person kick the ball correctly?

Exercises for nonaction-oriented BR

5) Puzzles
The participant is given the following instruction: “I am going to show you a picture of a person's body. Then, we will recreate it by assembling a series of pieces, just like solving a puzzle”.
For each session, 1 puzzle is proposed.

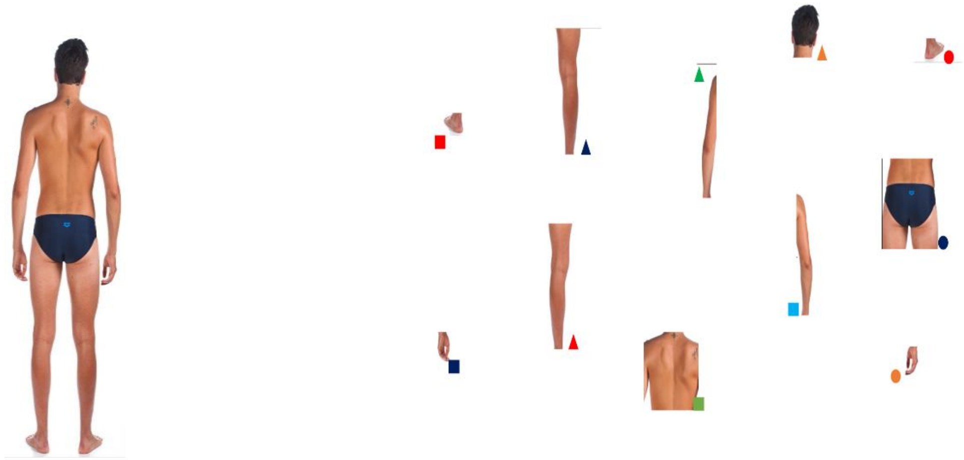

Figure S3. *Example of Puzzle with a reference model Exercise*.

6) Human Body with Misplaced or Disproportionate Parts
The participant is given the following instruction: “I am going to show you some pictures of body parts. You need to tell me whether you think each one is correct or incorrect. If you think a picture is incorrect, please explain what the error is”.
For each session, 2 different pictures are proposed.

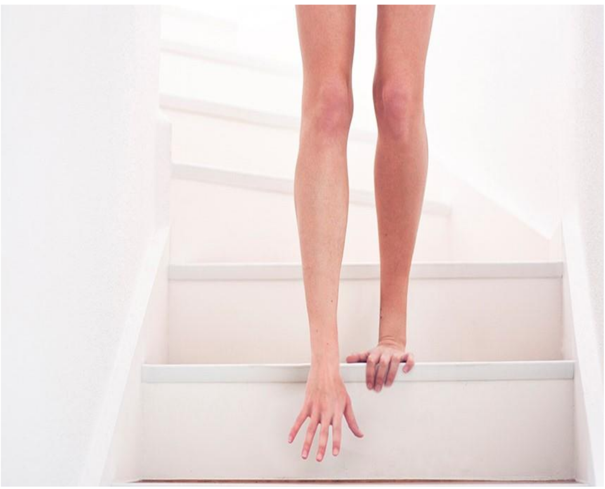

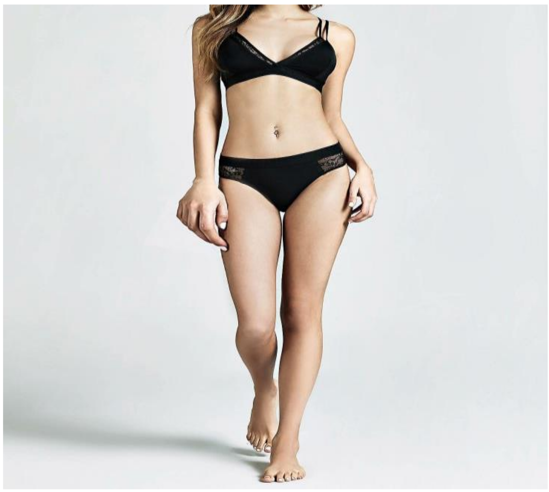

Figure S4. *Example of the Human Body with Misplaced (on the left) or Disproportionate Parts (on the right) Exercise.*

7) Wooden Mannequin Exercises

A) Matching patient-mannequin

The participant is given the following instruction: “I am now going to show you a wooden mannequin. Your task is to take on the same position as the mannequin”.

B) Matching of mannequin

The participant is given the following instruction: ”I am now going to show you two wooden mannequins. Your task is to tell me whether or not they assume the same posture”.

For each session, 4 different matchings are proposed (two in mode A and two in mode B).

Table S3. Example of Wooden Mannequin Exercise.

A) Matching patient-mannequin
Non-hemiparetic upward-facing arm
Non-hemiparetic arm straight along the body and pointing downwards
B) Matching of mannequin
Left leg bent back
Bust bent forward

8) The Body Dominoes

The participant is given the following instruction: “I am now going to show you tiles of dominoes representing different parts of the body or names of body parts (such as hand, foot, knee, etc.). You start by choosing a tile and placing it in the middle. Then, it is my turn to place a tile that corresponds to one of the body parts contiguous to the one previously placed in the centre (for example, the nose is contiguous to the mouth). We proceed in turns. Your task is to form a long row and match the tiles by considering the proximities of the body parts”.

For each session, 1 dominoes is proposed.

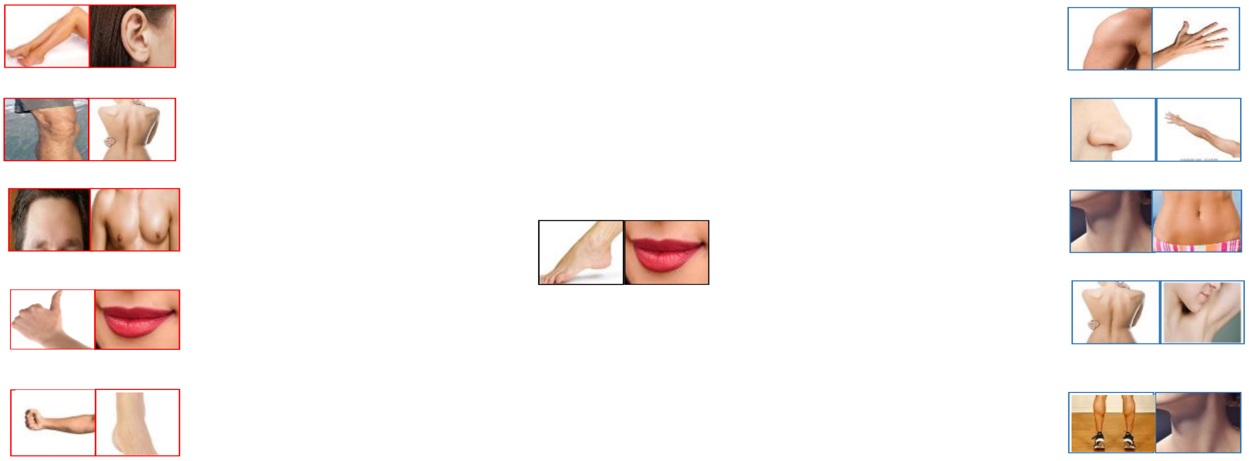

Figure S5. *Example of the Body Dominoes Exercise (picture version).*
